# Birth of a boy after intracytoplasmic sperm injection using ejaculated spermatozoa from a nonmosaic klinefelter syndrome man with normal sperm motility: A case report

**DOI:** 10.3389/fgene.2022.989701

**Published:** 2022-09-23

**Authors:** Wen-qing Xu, Yuan Yuan, Ying Chen, Tao Luo, Hou-yang Chen

**Affiliations:** ^1^ Institute of Life Science and School of Life Science, Nanchang University, Nanchang, China; ^2^ Key Laboratory of Reproductive Physiology and Pathology in Jiangxi Province, Nanchang, China; ^3^ Reproductive Medical Center, Jiangxi Maternal and Child Health Hospital, Affiliated Maternal and Child Health Hospital of Nanchang University, Nanchang, China

**Keywords:** nonmosaic klinefelter syndrome, male infertility, spermatogenesis, sperm motility, intracytoplasmic sperm injection

## Abstract

Klinefelter syndrome (KS) is the most common sex chromosome abnormality, which occurs in about one in 660 newly born males, and it is the most common genetic cause of infertility in infertile men, accounting for 11%. It is rare for non-mosaic KS patients to have sperm and reproduce naturally, and there are currently no reports of KS patients with normal motile sperm. Microdissection testicular sperm extraction associated with intracytoplasmic sperm injection (micro-TESE-ICSI) is currently the main assisted reproductive method for patients with KS. In this study, we describe a patient of non-mosaic KS (47, XXY) who had given birth to a healthy girl naturally. The patient had normal male characteristics and did not have the symptoms of hypogonadism commonly seen in KS. He had high levels of serum follicle stimulating hormone and luteinizing hormone, a low level of serum testosterone, and a normal level of prolactin. Semen analysis showed that this case had normal motile sperm (total motility of 57.66% and progressive motility of 46.19%) but low sperm concentration (1.7 × 10^6^ cells/mL). He gave birth to a boy by intracytoplasmic sperm injection (ICSI) using his ejaculated sperm purified to high density and motility by Percoll density gradient centrifugation. In conclusion, this case is a unique non-mosaic KS patient who had a normal sperm motility, experienced a natural fertility, and received a successful ICSI outcome, which enlarges our knowledges on non-mosaic KS.

## Introduction

Klinefelter’s syndrome (KS) is a common sex chromosome abnormality and occurs in about 0.10–0.15% of males (Bojesen et al., 2003). KS includes nonmosaic (47, XXY) and mosaic types; nonmosaic KS is the major type, accounting for about 90% of KS cases (Abdelmoula et al., 2004; Lanfranco et al., 2004; Paduch et al., 2008; Samplaski et al., 2014). KS is the most common genetic cause of male infertility (Kanakis and Nieschlag, 2018). Men with nonmosaic KS usually exhibit azoospermia, but less than 10% of patients have oligoasthenozoospermia and a small number of abnormal spermatozoa in their ejaculates (Staessen et al., 2003; Ulug et al., 2003; Ichioka et al., 2006). With the help of assisted reproductive technology, microdissection testicular sperm extraction associated with intracytoplasmic sperm injection (micro-TESE-ICSI) provides these KS patients with azoospermia the opportunity to have children. Several studies have reported that patients with KS could father a child with a normal peripheral blood karyotype by intracytoplasmic sperm injection (ICSI) using spermatozoa extracted from testicular biopsy samples (Nodar et al., 1999; Poulakis et al., 2001; Greco et al., 2013) or ejaculated spermatozoa (Ichioka et al., 2006). Although spermatozoa can be found in the semen ejaculated from a few patients with KS, there are no reports of normal sperm motility in patients with KS, nor have such patients ever given birth to a child with a normal karyotype naturally. This study presents the first case of a non-mosaic KS man who had previously given birth naturally, using ICSI to achieve fertility using ejaculated sperm with normal sperm motility.

## Case presentations

A 31-year-old man and his 29-year-old wife were admitted to the Reproductive Medicine Center of Jiangxi Maternal and Child Health Hospital affiliated with Nanchang University in 2018 for subfertility assessment. The couple participating in this study provided signed informed consent. This study was approved by the Institutional Ethics Committee on human subjects of Jiangxi Maternal and Child Health Hospital. The couple had been married for 8 years and had had a girl naturally in the second year of marriage. After 3 years of marriage, they wanted a second child, so they stopped using contraception, but had not succeeded in giving birth for 5 years. They had no significant medical or surgical history and were not on any long-term medications. The wife was checked to ensure that both fallopian tubes were unblocked, and her reproductive function was normal. Classic karyotyping of metaphases by Giemsa(G)-banding was performed as described elsewhere (Huang and Chen, 2017). G-banding chromosomal analysis showed that the wife and their first child had normal chromosomal karyotypes but the husband had a sex chromosome abnormality (47, XXY). The karyotype result of the patient was shown in Supplementary Figure S1. Y chromosome microdeletions were not detected in the husband. Therefore, the husband has nonmosaic KS.

The patient underwent andrology examination, semen analysis, and endocrine evaluation. The patient has a laryngeal knot and normal male breasts. The pubic hair is distributed in male characteristics, the penis developed normally, and the bilateral epididymis and vas deferens developed normally. According to testicular ultrasonography, the volumes of the left and right testes are calculated to be 6.6 ml and 7 ml, respectively (Supplementary Figure S2). Semen analysis revealed that the patient has a low number of spermatozoa in his ejaculates (Table 1). It is worth noting that the patient has normal sperm motility (Table 1). In addition, the patient has high levels of serum follicle stimulating hormone and luteinizing hormone, a low level of serum testosterone, and a normal level of prolactin (Table 1).

**TABLE 1:** Testicle volume, semen parameters, and hormone profile.

Parameters	Patient	References
Testis size		
Left (ml)	6.6	12–25
Right (ml)	7	12–25
Semen parameters		
Volume (ml)	2.0	1.5–6
pH	7.4	7.2–8
Sperm concentration (10^6^ cells/mL)	1.7	≥15
Sperm count (10^6^ cells)	3.4	≥39
Total motility (%)	57.66	≥40
Progressive motility (%)	46.19	≥32
Serum hormones		
FSH (mIU/L)	35.37	1.3–11.8
LH (mIU/L)	26.46	1.8–8.4
PRL (ng/ml)	10.16	4.04–15.2
T (ng/mL)	1.55	2.6–7.4

FSH, follicle-stimulating hormone; LH, luteinizing hormone; T, testosterone; PRL, prolactin.

Sufficient clinical evidence has found that patients with KS usually present with symptoms of hypogonadism such as tall height, small, hard testes, and disproportion between upper and lower body (Bonomi et al., 2017). Notably, this patient was 165 cm tall, weighed 60 kg, and a testicular volume of approximately 7 ml (Table 1) was not small for a man with an FSH of 35.37 mIU/L. Furthermore, the patient had normal male characteristics, which indicated that the patient did not have the symptoms of hypogonadism commonly seen in KS.

After consulting with the genetics department, we recommended the treatment of preimplantation genetic testing (PGT). The couple decided against PGT for economic reasons and insisted on ICSI. Because the husband’s sperm motility is normal, but the sperm concentration is low, Percoll density gradient centrifugation on his spermatozoa was performed to increase the sperm concentration. Seven metaphase II oocytes were retrieved for ICSI and four oocytes were fertilized. Three transplantable embryos formed after standard embryo culture and one high-quality embryo was transferred. Eleven days after embryo transplantation, beta-human chorionic gonadotropin was determined to be positive. The wife later gave birth to a boy.

## Discussion

The etiology of nonmosaic KS is an increase in the number of X chromosomes caused by errors during meiosis (Radicioni et al., 2010). The gene dosage effect caused by the increase in the number of X chromosomes leads to the cumulative effect of gene imbalance located in the trichromosomes or chromosomal regions, which is the main cause of male infertility symptoms in patients with KS (Scofield et al., 2008; Ottesen et al., 2010; Zhang et al., 2020). This effect may cause abnormal expression of spermatogenesis-related genes; hence, most patients with KS have azoospermia. However, some patients with KS still have sperm in their semen and they have varying degrees of a male phenotype. In this study, we report the first non-mosaic KS patient with sperm in ejaculate and normal sperm motility. This may be related to the silencing of spermatogenesis-related genes on the extra X chromosome, or additional methylation modifications, so that these genes still have partial functions. This finding suggests that by studying the corresponding methylation or unique genetic imprints on the extra X chromosome, it is possible to discover the mechanism by which spermatogenesis and motility are regulated in patients with KS.

TESE-ICSI is the main method of assisting the reproduction of patients with KS (Denschlag et al., 2004; Corona et al., 2017). Compared with TESE-ICSI, ICSI does not require sperm retrieval from the testis and is less invasive to the patient. And using the patient’s ejaculated sperm for ICSI can give patients more confidence in the success rate of the procedure. In the present study, we report the first case of a non-mosaic KS patient who had previously given birth naturally and successfully conceived with ICSI using his normally motile ejaculate sperm, providing additional clinical data for addressing fertility problems in non-mosaic KS patients. This case suggests to us that studying the mechanism of action at different phenotypic levels in KS patients will help to develop drugs to improve sperm quality in these patients, improving fertility and reproductive quality.

In both the case presented and another case reported by Ichioka et al. (2006), the outcome was natural childbearing and developed oligospermia over time. This indicates that the semen quality of patients with KS will gradually decline. Estimates from epidemiological data suggest that the prevalence of KS is higher than the number of patients who are clinically diagnosed (Bojesen et al., 2003). It shows that many KS patients have not really received clinical diagnosis, let alone screened out in adolescence. Therefore, for KS patients, early popularization of chromosome screening technology and early intervention is meaningful. The use of testosterone replacement therapy and other treatments can effectively improve the quality of life of KS patients and change reproductive outcomes (Rives et al., 2013; Plotton et al., 2014).

Another notable thing in this case is that the patient did not have typical symptoms of hypogonadism. The clinical features depend on the extra X chromosome and the effects of hypogonadism (Visootsak and Graham, 2006). However, what we know about the signs and symptoms of KS is only the tip of the iceberg. Unfortunately, what we know about the clinical presentation of men with KS comes directly from descriptions of men who have received a specific diagnosis of KS (Lanfranco et al., 2004). This case illustrates that not all patients with KS have typical symptoms of hypogonadism, which may be related to the extra X chromosome not functioning. Therefore, the symptoms of hypogonadism of men cannot be used to adequately judge whether it is a patient with KS and its severity.

This study reports the first case of a non-mosaic KS male who had ever given birth naturally, a boy born after ICSI using his normally motile ejaculate sperm. This case shows that sperm quality in KS patients declines over time. And hypogonadism is not the defining symptom of KS. This case reminds the study of the effect of extra X chromosome inactivation on spermatogenesis and sperm motility in KS patients, provides more clinical data for KS assisted reproductive therapy, and reminded that the early intervention treatment of KS patients may be a new idea to help future fertility.

## Data Availability

The raw data supporting the conclusions of this article will be made available by the authors, without undue reservation.

## Supplementary material

SUPPLEMENTARY FIGURE S1 Karyotype result of the patient. The G-banding method was used to detect the karyotype, and the patient's karyotype was 47, XXY.

Supplementary Figure S2 Testicular B-ultrasound pictures and volume calculation results of the patient. The formula for calculating testis volume from b-ultrasound images is 0.71 × length × width × height. Using this formula, we calculated that the volumes of the patient's left and right testes were 6.6 ml and 7 ml, respectively.

## References

[B1] Abdelmoula N. B. Amouri A. Portnoi M. F. Saad A. Boudawara T. Mhiri M. N. (2004). Cytogenetics and fluorescence *in situ* hybridization assessment of sex-chromosome mosaicism in Klinefelter's syndrome. Ann. Genet. 47 (2), 163–175. 10.1016/j.anngen.2003.08.024 15183749

[B2] Bojesen A. Juul S. Gravholt C. H. (2003). Prenatal and postnatal prevalence of klinefelter syndrome: a national registry study. J. Clin. Endocrinol. Metab. 88 (2), 622–626. 10.1210/jc.2002-021491 12574191

[B3] Bonomi M. Rochira V. Pasquali D. Balercia G. Jannini E. A. Ferlin A. (2017). Klinefelter syndrome (KS): genetics, clinical phenotype and hypogonadism. J. Endocrinol. Invest. 40 (2), 123–134. 10.1007/s40618-016-0541-6 27644703 PMC5269463

[B4] Corona G. Pizzocaro A. Lanfranco F. Garolla A. Pelliccione F. Vignozzi L. (2017). Sperm recovery and ICSI outcomes in klinefelter syndrome: a systematic review and meta-analysis. Hum. Reprod. Update 23 (3), 265–275. 10.1093/humupd/dmx008 28379559

[B5] Denschlag D. Tempfer C. Kunze M. Wolff G. Keck C. (2004). Assisted reproductive techniques in patients with klinefelter syndrome: a critical review. Fertil. Steril. 82 (4), 775–779. 10.1016/j.fertnstert.2003.09.085 15482743

[B6] Greco E. Scarselli F. Minasi M. G. Casciani V. Zavaglia D. Dente D. (2013). Birth of 16 healthy children after ICSI in cases of nonmosaic Klinefelter syndrome. Hum. Reprod. 28 (5), 1155–1160. 10.1093/humrep/det046 23493114

[B7] Huang H. Chen J. (2017). Chromosome bandings. Methods Mol. Biol. 1541, 59–66. 10.1007/978-1-4939-6703-2_6 27910014

[B8] Ichioka K. Utsunomiya N. Kohei N. Ueda N. Inoue K. Terai A. (2006). Adult onset of declining spermatogenesis in a man with nonmosaic Klinefelter's syndrome. Fertil. Steril. 85 (5), 1511 e1511–e2. 10.1016/j.fertnstert.2005.10.069 16616747

[B9] Kanakis G. A. Nieschlag E. (2018). Klinefelter syndrome: more than hypogonadism. Metabolism. 86, 135–144. 10.1016/j.metabol.2017.09.017 29382506

[B10] Lanfranco F. Kamischke A. Zitzmann M. Nieschlag E. (2004). Klinefelter's syndrome. Lancet 364 (9430), 273–283. 10.1016/S0140-6736(04)16678-6 15262106

[B11] Nodar F. De Vincentiis S. Olmedo S. B. Papier S. Urrutia F. Acosta A. A. (1999). Birth of twin males with normal karyotype after intracytoplasmic sperm injection with use of testicular spermatozoa from a nonmosaic patient with Klinefelter's syndrome. Fertil. Steril. 71 (6), 1149–1152. 10.1016/s0015-0282(99)00151-x 10360927

[B12] Ottesen A. M. Aksglaede L. Garn I. Tartaglia N. Tassone F. Gravholt C. H. (2010). Increased number of sex chromosomes affects height in a nonlinear fashion: a study of 305 patients with sex chromosome aneuploidy. Am. J. Med. Genet. A 152A (5), 1206–1212. 10.1002/ajmg.a.33334 20425825 PMC5454803

[B13] Paduch D. A. Fine R. G. Bolyakov A. Kiper J. (2008). New concepts in Klinefelter syndrome. Curr. Opin. Urol. 18 (6), 621–627. 10.1097/MOU.0b013e32831367c7 18832949

[B14] Plotton I. Brosse A. Cuzin B. Lejeune H. (2014). Klinefelter syndrome and TESE-ICSI. Ann. Endocrinol. 75 (2), 118–125. 10.1016/j.ando.2014.04.004 24786702

[B15] Poulakis V. Witzsch U. Diehl W. de Vries R. Becht E. Trotnow S. (2001). Birth of two infants with normal karyotype after intracytoplasmic injection of sperm obtained by testicular extraction from two men with nonmosaic Klinefelter's syndrome. Fertil. Steril. 76 (5), 1060–1062. 10.1016/s0015-0282(01)02830-8 11704137

[B16] Radicioni A. F. Ferlin A. Balercia G. Pasquali D. Vignozzi L. Maggi M. (2010). Consensus statement on diagnosis and clinical management of Klinefelter syndrome. J. Endocrinol. Invest. 33 (11), 839–850. 10.1007/BF03350351 21293172

[B17] Rives N. Milazzo J. P. Perdrix A. Castanet M. Joly-Helas G. Sibert L. (2013). The feasibility of fertility preservation in adolescents with Klinefelter syndrome. Hum. Reprod. 28 (6), 1468–1479. 10.1093/humrep/det084 23539613

[B18] Samplaski M. K. Lo K. C. Grober E. D. Millar A. Dimitromanolakis A. Jarvi K. A. (2014). Phenotypic differences in mosaic Klinefelter patients as compared with non-mosaic Klinefelter patients. Fertil. Steril. 101 (4), 950–955. 10.1016/j.fertnstert.2013.12.051 24502895

[B19] Scofield R. H. Bruner G. R. Namjou B. Kimberly R. P. Ramsey-Goldman R. Petri M. (2008). Klinefelter's syndrome (47, XXY) in male systemic lupus erythematosus patients: support for the notion of a gene-dose effect from the X chromosome. Arthritis Rheum. 58 (8), 2511–2517. 10.1002/art.23701 18668569 PMC2824898

[B20] Staessen C. Tournaye H. Van Assche E. Michiels A. Van Landuyt L. Devroey P. (2003). PGD in 47, XXY Klinefelter's syndrome patients. Hum. Reprod. Update 9 (4), 319–330. 10.1093/humupd/dmg029 12926526

[B21] Ulug U. Bener F. Akman M. A. Bahceci M. (2003). Partners of men with Klinefelter syndrome can benefit from assisted reproductive technologies. Fertil. Steril. 80 (4), 903–906. 10.1016/s0015-0282(03)01157-9 14556810

[B22] Visootsak J. Graham J. M. Jr. (2006). Klinefelter syndrome and other sex chromosomal aneuploidies. Orphanet J. Rare Dis. 1, 42. 10.1186/1750-1172-1-42 17062147 PMC1634840

[B23] Zhang X. Hong D. Ma S. Ward T. Ho M. Pattni R. (2020). Integrated functional genomic analyses of Klinefelter and Turner syndromes reveal global network effects of altered X chromosome dosage. Proc. Natl. Acad. Sci. U. S. A. 117 (9), 4864–4873. 10.1073/pnas.1910003117 32071206 PMC7060706

