# Supplementary figures and images for: Birth of a boy after intracytoplasmic sperm injection using ejaculated spermatozoa from a nonmosaic klinefelter syndrome man with normal sperm motility: A case report
Source: Front Genet. 2022 Sep 23;13:989701. doi: 10.3389/fgene.2022.989701 (PMC9538340; doi:10.3389/fgene.2022.989701)

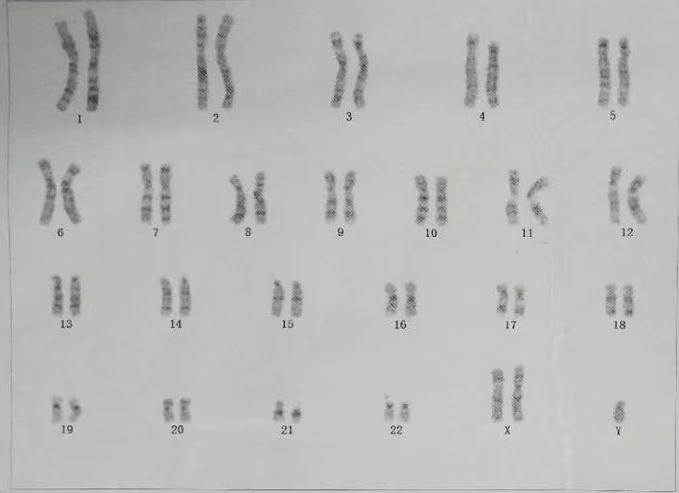

Supplement: Supplementary file 1 [file Image1.JPEG]

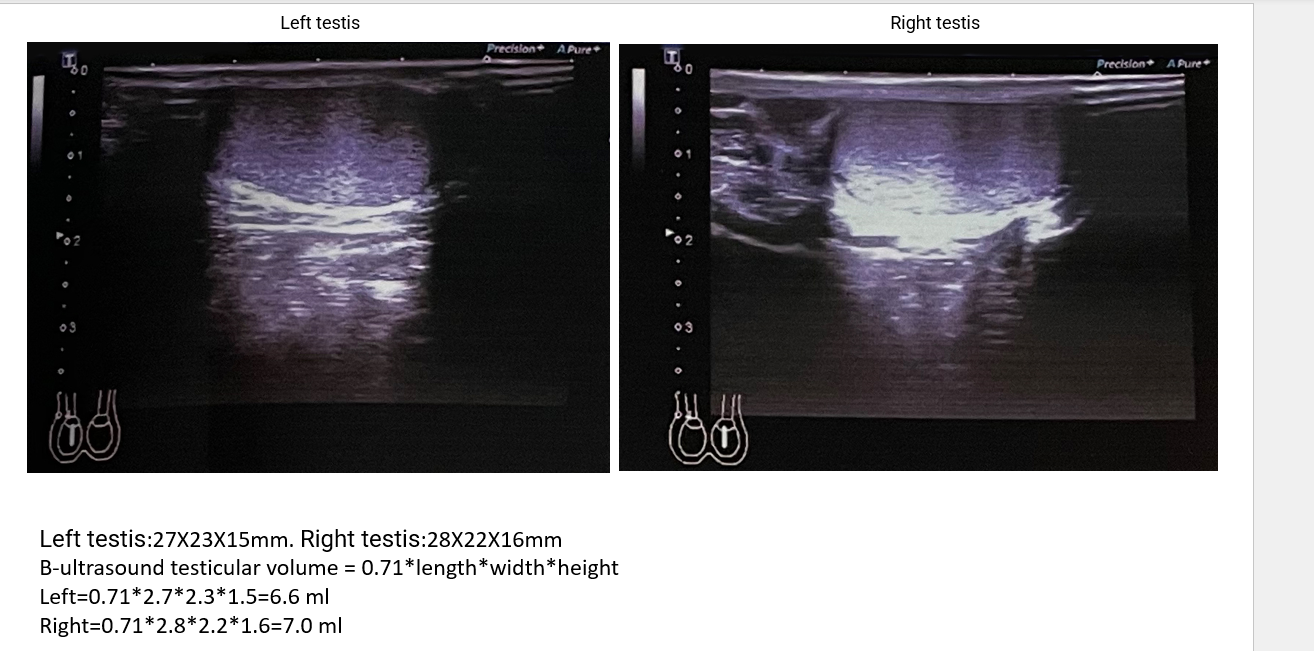

Supplement: Supplementary file 2 [file Image2.PNG]
